# Phosphorylation Regulates SIRT1 Function

**DOI:** 10.1371/journal.pone.0004020

**Published:** 2008-12-24

**Authors:** Tsutomu Sasaki, Bernhard Maier, Katarzyna D. Koclega, Maksymilian Chruszcz, Wendy Gluba, P. Todd Stukenberg, Wladek Minor, Heidi Scrable

**Affiliations:** 1 Neuroscience Graduate Program, University of Virginia, Charlottesville, Virginia, United States of America; 2 Department of Neuroscience, University of Virginia, Charlottesville, Virginia, United States of America; 3 Department of Molecular Physiology and Biophysics, University of Virginia, Charlottesville, Virginia, United States of America; 4 Department of Biochemistry and Molecular Genetics, University of Virginia, Charlottesville, Virginia, United States of America; University of Minnesota, United States of America

## Abstract

**Background:**

*SIR2* is an NAD^+^-dependent deacetylase [1]–[3] implicated in the regulation of lifespan in species as diverse as yeast [4], worms [5], and flies [6]. We previously reported that the level of SIRT1, the mammalian homologue of SIR2 [7], [8], is coupled to the level of mitotic activity in cells both *in vitro* and *in vivo* [9]. Cells from long-lived mice maintained SIRT1 levels of young mice in tissues that undergo continuous cell replacement by proliferating stem cells. Changes in SIRT1 protein level were not associated with changes in mRNA level, suggesting that SIRT1 could be regulated post-transcriptionally. However, other than a recent report on sumoylation [10] and identification of SIRT1 as a nuclear phospho-protein by mass spectrometry [11], post-translational modifications of this important protein have not been reported.

**Methodology/Principal Findings:**

We identified 13 residues in SIRT1 that are phosphorylated *in vivo* using mass spectrometry. Dephosphorylation by phosphatases *in vitro* resulted in decreased NAD^+^-dependent deacetylase activity. We identified cyclinB/Cdk1 as a cell cycle-dependent kinase that forms a complex with and phosphorylates SIRT1. Mutation of two residues phosphorylated by Cyclin B/Cdk1 (threonine 530 and serine 540) disturbs normal cell cycle progression and fails to rescue proliferation defects in SIRT1-deficient cells [12], [13].

**Conclusions/Significance:**

Pharmacological manipulation of SIRT1 activity is currently being tested as a means of extending lifespan in mammals. Treatment of obese mice with resveratrol, a pharmacological activator of SIRT1, modestly but significantly improved longevity and, perhaps more importantly, offered some protection against the development of type 2 diabetes mellitus and metabolic syndrome [14]–[16]. Understanding the endogenous mechanisms that regulate the level and activity of SIRT1, therefore, has obvious relevance to human health and disease. Our results identify phosphorylation by cell cycle dependent kinases as a major mechanism controlling the level and function of this sirtuin and complement recent reports of factors that inhibit [17], [18] and activate [19] SIRT1 by protein-protein interactions.

## Introduction

The *SIR2* gene encodes an NAD^+^-dependent deacetylase [1]–[3]. It was first identified in yeast as a gene involved in mating type switching [20], but is now known to be a highly conserved gene in organisms ranging from archea to humans [21]. Of the seven *SIR2* family homologues (sirtuins) in humans [7], [8], *SIRT1* is most closely related to the *SIR2* gene of *Saccharomyces cerevisiae* [8]. Over-expression of *SIR2* extends replicative life-span in yeast [4], and orthologs extend organismal life-span in both worms and flies [5], [6]. Recently, it was shown that resveratrol, a pharmacological activator of SIRT1, can improve the life span and health of mice on a typical “western” (high-calorie) diet [15], [16].

We previously reported that the level of SIRT1 is coupled to the level of mitotic activity in cells both *in vitro* and *in vivo* [9]. Changes in SIRT1 protein level were not associated with changes in mRNA level, suggesting that SIRT1 could be regulated post-transcriptionally. However, other than phosphorylation of serine 27 and serine 47, which were detected in general screens of nuclear phospho-proteins by mass spectrometry [11] and sumoylation of lysine 734 [10], post-translational modifications of this important protein have not been reported.

In this work, we investigated the connection between SIRT1 protein levels and mitotic activity by determining if there was a direct effect of mitotic cell cycle kinases on SIRT1 phosphorylation. In somatic cells, cyclin D/Cdk 4,6 is active during the progression through G_1_ and into S phase. Cyclin E/Cdk 2 complex becomes active at late G_1_ phase into S phase. CyclinA/Cdk2 becomes active during S phase, and the CyclinB/Cdk1 complex is activated upon passing the G_2_/M checkpoint and inactivated upon entry into anaphase [22]. We also explored the hypothesis that phosphorylation might regulate the deacetylase activity of SIRT1, as it is known to do with other classes of protein deacetylases, such as HDAC1 and HDAC2 [23], [24]. As described below, we found that SIRT1 is phosphorylated by cyclinB/Cdk1, and that phosphorylation regulates its deacetylase activity and affects cell proliferation.

## Results

### SIRT1 is phosphorylated at 13 residues *in vivo*

**Figure 1:**
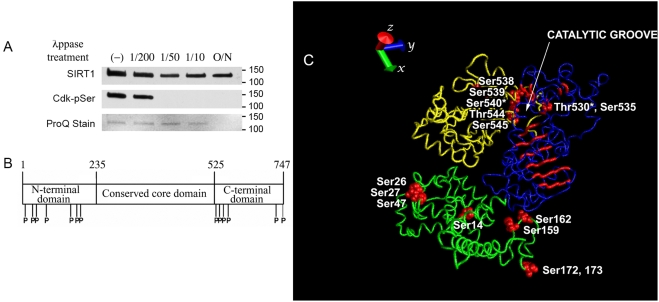
SIRT1 is a phosphoprotein. A. Affinity-purified SIRT1 is phosphorylated, and can be dephosphorylated by λppase. Top panel, western blot of total SIRT1; middle panel, western blot of SIRT1 phosphorylated at the Cdk consensus site; bottom panel, ProQ-diamond stained blot showing total phosphorylated SIRT1. Numbers above the lanes indicate the relative doses of λppase used to treat FLAG-SIRT1 for 1 hour. Overnight treatment (O/N) was done with a 1/10 dilution of λppase. B. Schematic of the phosphorylated residues in SIRT1 identified by mass spectrometry, as shown in Table 1. P, phosphorylated residue. C. 3D structure prediction of full-length human SIRT1 with the positions of phosphorylated residues identified by mass spectrometry (red symbols). Amino terminal domain, green; catalytic core domain, blue; carboxy terminal domain, yellow. The Rossmann fold supporting the catalytic groove in the core domain is shown in red. The two mutated residues described in the text (Thr530 and Ser540) are indicated with asterisks.

To determine if SIRT1 is a phosphoprotein, we stained gels containing affinity-purified FLAG-SIRT1 separated by SDS-PAGE with Pro-Q Diamond phosphoprotein reagent. We also performed western analysis using an antibody that detects the phosphorylated serine residue in the consensus Cdk recognition motif (K/R-S*-P-x-K/R). As shown in **Fig. 1A**, both the anti-phospho serine Cdk substrate antibody and the ProQ reagent detect a protein that migrates to the same position in the gel as FLAG-SIRT1 (120 kD; lane marked “-”). The signals decreased in a dose-dependent manner following treatment with lambda protein phosphatase (λppase). Although reaction with the anti- phospho serine Cdk substrate antibody was lost at a low dose of λppase, some reactivity with the phosphoprotein stain, which detects all phospho-residues, was still visible even after treatment with high doses of λppase and required overnight treatment for complete removal. This difference in sensitivity to λppase treatment of the two detection methods implied that SIRT1 could be phosphorylated at multiple residues, and could contain phospho-threonine and phospho-tyrosine residues in addition to phospho-serine.

To identify the residues of SIRT1 that are phosphorylated *in vivo*, we analyzed affinity-purified FLAG-SIRT1 by mass spectrometry. The analysis revealed 13 phosphorylation sites (**Table 1**), including threonine 530 and serine 540. Phosphorylation was restricted to serine and threonine residues; there were no phosphorylated tyrosine residues in the samples examined. All of the identified phosphorylation sites are located in either the N-terminal domain or the C-terminal domain of SIRT1, and not in the conserved catalytic core domain (**Fig. 1B**). To illustrate how the phosphorylation sites could potentially regulate substrate access and protein interactions, we used the known amino acid sequence of SIRT to derive a full chain protein structure prediction based on homology and *ab initio* modeling using the Robetta server (Baker Laboratory, http://robetta.bakerlab.org/). As shown in **Fig. 1C**, the structure predicts an extensive clustering of phosphorylation sites on residues in the carboxy terminal domain (yellow) adjacent to the Rossmann fold (red) that supports the catalytic groove in the core domain (blue). Phosphorylation of residues in the amino terminal domain (green) are less likely to be directly involved in regulating enzyme activity.

**Table 1:** Phospho-peptide mapping of FLAG-SIRT1 by mass spectrometry.

Sequence of Identified Peptides	Sites
MADEAALALQPGGS*PSAAGADR	S14
EAAS*SPAGEPLR	S26
EAASS*PAGEPLR	S27
MADEAALALQPGGS*PSAAGADREAAS?S?PAGEPLR	S14, and S26 or S27
S*PGEPGGAAPER	S47
DNLLFGDEIITNGFHS?CES?DEEDR	S159 or S162
ASHASS*SDWTPRPR	S173
ASHAS*S*SDWTPRPR	S172 and S173 likely (2 out of 3 sites within S172–S174)
ELAYLSELPPT*PLHVSEDSSSPER	T530
ELAYLSELPPTPLHVSEDS?S?S?PER	1 of 3 sites within S538–S540
ELAYLSELPPTPLHVS?EDS?S?S?PER	2 of 4 sites within S535 and S538–S540
T?S?PPDSSVIVTLLDQAAK	T544 or S545
AGGAGFGT*DGDDQEAINEAISVK	T719
QEVTDMNYPSNKS*	S747

Phosphorylation is indicated by an asterisk (*). A site that could not be ruled out is indicated by a question mark (?). Two independent samples of affinity-purified FLAG-SIRT1 were analyzed. The results were consistent between the two, except for S747 phosphorylation, which was detected in only one of the samples.

### Cyclin recognition motifs and Cdk substrate residues are conserved among orthologs of SIRT1

To determine if the phosphorylation sites identified by mass spectrometry were evolutionarily conserved, we compared the amino acid sequences surrounding each phosphorylated residue in 12 different species (**Table 2**). Among the phosphorylated residues listed in **Table 1**, Ser159, Ser162, Ser172, Ser173, Thr530, Ser535, Ser538, and Ser540 are relatively well conserved. A cyclin recognition motif and a Cdk substrate motif together constitute a bipartite substrate recognition sequence for cyclin-dependent kinases [25]. Ser540 fits the full consensus sequence for a Cdk substrate (S/T*-P-x-K/R), and Thr530 fits the minimal consensus sequence for a Cdk substrate (S/T*-P) [26]–[28]. Two cyclin recognition motifs (located between amino acid 203–207 and 519–523) are also well conserved. Some of the phosphorylation sites are not conserved because the N-terminal and the C-terminal domains are not present in orthologs of SIRT1 in lower organisms. Other sites that fit the minimal consensus sequence for Cdk substrates, such as Ser14, Ser26/27, Ser47 and Ser545, are only conserved in higher organisms.

**Table 2:** Conservation of phosphorylated residues, cyclin recognition motifs, and Cdk substrate motifs among SIRT1 orthologs of 12 different species.

Species	14	15	26	27	28	47	48	159	162	172	173	cyc 203–207	cyc 519–523	530	531	535	538	539	540	Cdk 537–543	544	545	546	719	747
*H.sapiens*	S	P	S	S	P	S	P	S	S	S	S	KDLLP	KELAY	T	P	S	S	S	S	DSS**S** PER	T	S	P	T	S
*P. troglodytes*	S	P	S	S	P	S	P	S	S	S	S	KDLLP	KELAY	T	P	S	S	S	S	DSS**S** PER	T	S	P	T	S
*M. mulatta*	S	P	S	P	P	S	P	S	S	S	S	KDDLP	KELAY	T	P	S	S	S	S	DSS**S** PER	T	S	P	T	S
*C. familiaris*	S	P	S	P	P	S	P	S	S	S	S	KDDLP	KELAH(?)	T	P	S	S	S	S	DSS**S** PER	T	S	P	I	S
***B. Taurus***	S	P	S	P	P	S	S	S	S	S	S	KDLLP	KELAH(?)	T	P	S	S	S	S	GSS**S** PER	T	S	P	A	S
***M. musculus***	S	P	S	Q	P	S	P	S	S	S	S	KDLLP	KELV(ok)	T	P	S	S	S	S	DSS**S** PER	T	V	P	A	S
***R. norvegicus***	L	G	A	G	R	D	P	S	S	S	S	KDLLP	KELV(ok)	T	P	S	S	S	S	DSS**S** PER	T	V	P	A	S
***G. gallus***	G	P	#	A	E	E	D	S	S	S	S	KDLLP	KELEM(ok)	T	P	S	S	G	S	DSG**S** PEQ	M	T	P	E	#
***D. melanogaster***	#	#	#	#	#	#	#	G	N	S	S	ASIMP(ng)	KELMP(ok)	#	#	R	S	S	E	CSS**E**SER	Q	S	Q	#	#
***C. elegans***	#	#	#	#	#	#	#	#	#	#	#	QQIFP(ng)	RQLI(ok)	I	C	N	S	S	D	DSS**D**EPT	L	K	K	#	#
***S. pombe***	#	#	#	#	#	#	#	#	S	N	I	KKLGI(ok)	#	#	#	#	#	#	#	#	#	#	#	#	#
***S. cerevisiae***	#	#	#	#	#	#	#	#	#	#	#	#	#	#	#	#	#	#	#	#	#	#	#	#	#

Comparison of SIRT1 sequences by “BLAST 2 Sequence” among species, in which the protein sequence of the SIRT1 ortholog was available. Listed in order of the similarity to human SIRT1, from top to bottom. For species for which ELM motif data were available (BOLD), motif conservation was analyzed as well. For cyclin recognition motif, “ok” stands for a complete conservation of the motif, “?” for a partial conservation, and “ng” for loss of conservation. SP (14–15, 26–28, 47–48, 545–546) and TP (530–531) fit the minimal Cdk substrate motif, and the sequence between amino acids 537–543 fits the full Cdk substrate motif.

Abbreviations: cyc, cyclin recognition motif; Cdk, full Cdk-substrate motif.

### SIRT1 deacetylase activity is modulated by phosphorylation

**Figure 2:**
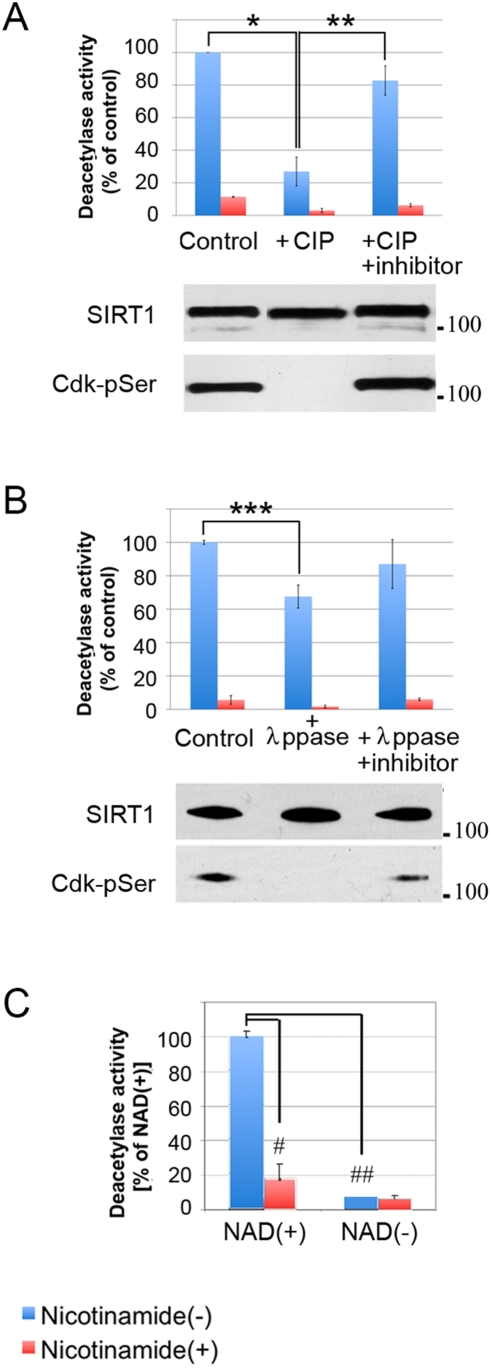
NAD^+^-dependent deacetylase activity of SIRT1 is modulated by phosphorylation. A/B. NAD^+^-dependent deacetylase activity of SIRT1 in the presence of CIP (A) or λppase (B) with or without phosphatase inhibitors. Western blots below each bar graph indicate the amount of SIRT1 in the reaction (top panel) and the degree of SIRT1 phosphorylation represented by Cdk-pSer signal (bottom panel). C. Deacetylase activity of affinity-purified FLAG-SIRT1 in the presence (left) or absence (right) of NAD^+^. Blue bars, no nicotinamide added; red bars, + nicotinamide. *, p<0.005; **, p<0.02; ***, p<0.002; #, p<0.01; ##, p<0.001 (Student's *t*-test). Error bars indicate +/−SEM.

To test the possibility that the phosphorylation of SIRT1 might modulate its NAD^+^-dependent deacetylase activity, we compared the enzymatic activity of phosphorylated and dephosphorylated affinity-purified FLAG-SIRT1 using a fluorogenic peptide-substrate-based assay system, *Fluor-de Lys* [29]. Dephosphorylation by calf intestinal phosphatase (CIP; **Fig. 2A**) or lambda phosphatase (λppase; **Fig. 2B**) led to a decline in deacetylase activity. This decline was associated with complete dephosphorylation at the Cdk sites in SIRT1, as shown in the accompanying images of western blots analyzed with an antibody against phosphorylated Cdk recognition sites. Neither the deacetylase activity nor the reaction with the anti-phosphoserine Cdk substrate antibody decreased when phosphatase activities were blocked (lanes marked “+inhibitor”). Therefore, the general loss of SIRT1 phosphorylation leads to a decline in its deacetylase activity. To rule out the possibility that changes in deacetylase activity were due to the activity of other classes of histone deacetylases, which are NAD^+^-independent, contaminating the FLAG-SIRT1 preparation, we repeated the *Fluor-de Lys* deacetylase assay in the absence of NAD^+^. As shown in **Fig. 2C**, there was no measurable deacetylase activity in the FLAG-SIRT1 preparation unless NAD^+^ was added to the reaction.

### SIRT1 forms a complex with cyclinB and Cdk1 *in vivo*

**Figure 3:**
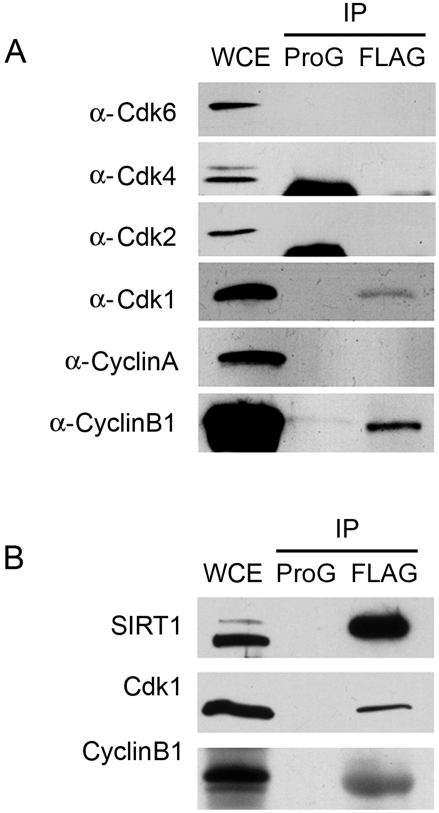
SIRT1 forms a complex with cyclinB/Cdk1. A. Western blot analysis of different Cdks and Cyclins following co-IP with FLAG-SIRT1. Complex formations of FLAG-SIRT1 with Cdk1 and cyclinB were detected, but not with Cdk2, Cdk4, Cdk6, or cyclinA. Amounts of samples used: whole cell extract (WCE), 20 µg; IP 500 µg. B. Western blot of co-IP experiments with endogenous SIRT1, which also forms a complex with Cdk1 and cyclinB. Amounts of samples used: WCE, 20 µg; IP 1.5 mg. WCE, whole cell extracts; IP, immunoprecipitation; FLAG, FLAG-M2 beads.

To determine if the highly conserved cyclin/Cdk substrate motifs at Thr530 and Ser540, which we found to be phosphorylated *in vivo*, are substrates for the enzyme, we transfected 293T cells with a FLAG-SIRT1 expression vector and prepared cell lysates from cells in exponential growth phase. We immunoprecipitated SIRT1 using anti-FLAG M2 beads and analyzed the immune complexes by western blot. We found Cdk1 in immune complexes with FLAG-SIRT1 (**Fig. 3A**). Cdk1 is a G2/M phase kinase that interacts with cyclinA and cyclinB to regulate mitosis [22]. We found that cyclinB, but not cyclinA, was detectable in a complex with FLAG-SIRT1 in asynchronously growing cultures (**Fig. 3A**). This interaction was specific, as no cyclin or Cdk proteins were immunoprecipitated when non-specific protein-G beads were substituted for the FLAG M2-specific beads (**Fig. 3A**, lanes marked “ProG”). The relatively low percentage of cyclinB detected in immune complexes reflects the relatively small number of cells in M-phase in these cultures, which could account for our inability to detect cyclinA in immune complexes with SIRT1 and Cdk1 as well. It is also possible that G1-associated kinases, such as Cdk2, Cdk4 and Cdk6, interact with SIRT1 at levels we could not detect, but this is less likely due to the relative abundance of cells in G1even in asynchronized cultures. Next, we performed co-immunoprecipitations from extracts of embryonic stem cells (ESCs) to determine if complexes between SIRT1 and Cdk1 and cyclinB also formed between endogenous proteins. As shown in **Fig. 3B**, antibodies against both N- and C-terminal epitopes of SIRT1 precipitated endogenous Cdk1 and cyclinB along with endogenous Sirt1 in ESCs. This interaction was specific, as no proteins were immunoprecipitated when non-specific protein-G beads were substituted for the FLAG M2-specific beads (**Fig. 3B**, lanes marked “ProG”). Compared to MEFs, a higher proportion of cyclinB was co-precipitated with SIRT1, reflecting the high proportion of ESCs that are in M-phase even in asynchronously growing cultures.

### SIRT1 is phosphorylated by cyclinB/Cdk1 *in vitro*

**Figure 4:**
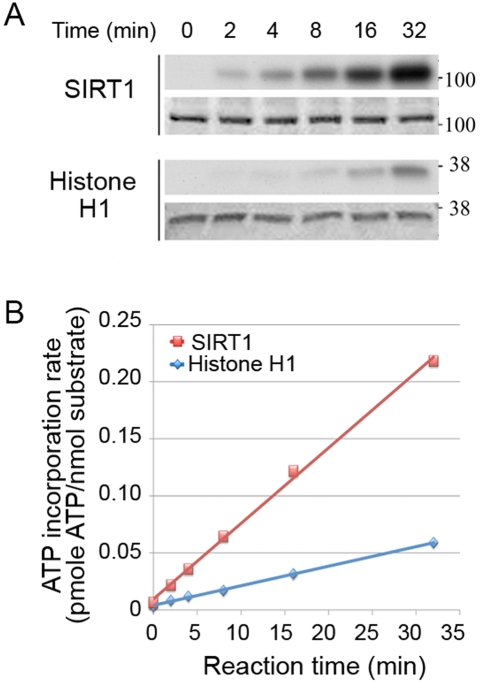
SIRT1 is phosphorylated by cyclinB/Cdk1 in vitro. A. Autoradiographs of radioactively labeled proteins separated by SDS-PAGE (upper panels) and pictures of Coomassie blue-stained gels (lower panels). Substrates are indicated to the left of each pair of panels. B. ATP incorporation *vs.* time. ATP incorporation was determined by scintillation counting of the radioactivity in each band.

**Figure 5:**
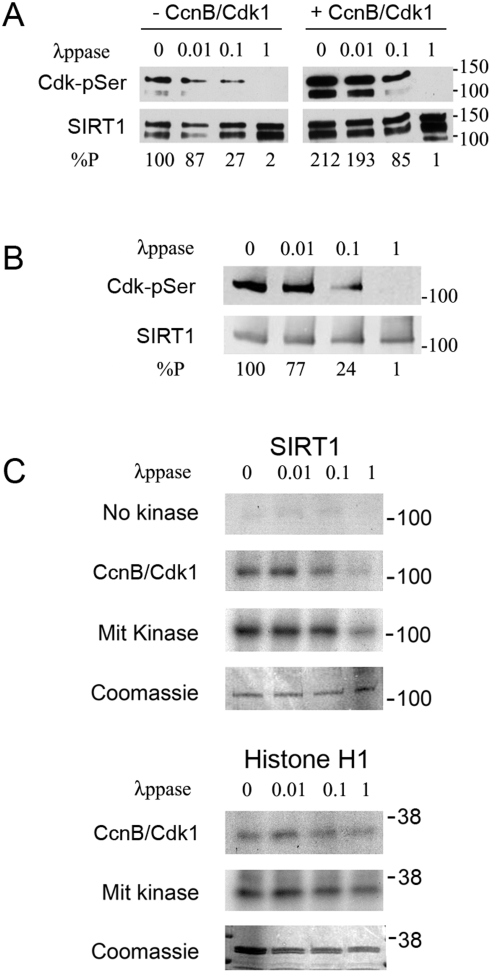
SIRT1 requires phosphorylation to become a suitable substrate for cyclinB/Cdk1 in vitro. A. Western blots of Cdk-phosphorylated (top panel) and total SIRT1 (bottom panel) following treatment with increasing doses of lambda phosphatase (λppase). The upper band corresponds to the full-length SIRT1. B. Levels of SIRT1 phosphorylation at Cdk-specific sites in extracts used as substrates for *in vitro* kinase assays shown in C. C. Autoradiographs (top panels) and Coomassie blue-stained gels (bottom panels) of SIRT1 and histone H1 phosphorylated by the kinases indicated on left. 0, 0.01, 0.1, 1, relative doses of λppase used; %P, relative percentage of Cdk phospho-serine signal compared to control.

To determine if cyclinB/Cdk1 could phosphorylate SIRT1, we performed *in vitro* kinase assays with affinity-purified human FLAG-SIRT1, using histone H1, a known substrate for cyclinB/Cdk1, as a positive control. We found that SIRT1 is an excellent substrate for phosphorylation by cyclinB/Cdk1 *in vitro* (**Fig. 4A–B**). We think that endogenous SIRT1 is also phosphorylated by this cyclin/cdk complex, although the reagents necessary to verify this experimentally are currently not available. Recombinant SIRT1, on the other hand, was a poor substrate (data not shown), probably reflecting a requirement for second-site phosphorylation, which would have been missing in this bacterially produced protein. To test the requirement for second-site phosphorylation directly, we subjected affinity-purified FLAG-SIRT1 to global dephosphorylation, then treated the dephosphorylated substrates with cyclinB/Cdk1. Phosphorylation at Cdk recognition sites in SIRT1 decreased as the dose of λppase increased, and was abolished when SIRT1 was maximally dephosphorylated (**Fig. 5A**). The level of phosphorylation at Cdk sites was higher in extracts with added cyclinB/Cdk1 than in extracts with no added kinase (compare left- and right-hand panels). Next, we performed a second *in vitro* kinase assay using radioactive ATP to detect protein phosphorylation by cyclinB/Cdk1. After treatment with higher doses of λppase, there was minimal incorporation of radioactive phosphate into SIRT1 (**Fig. 5C**). Histone H1, however, which is not affected by second-site phosphorylation, was efficiently labeled by radioactive ATP even at the highest doses of λppase. These data imply that additional phosphorylation sites increase the suitability of SIRT1 as a substrate for cyclinB/Cdk1. To test this, we used a mitotic kinase mix, which contains high levels of several mitotic-phase kinases in addition to cyclinB/Cdk1 [30], to phosphorylate FLAG-SIRT1. In the presence of these additional kinases, SIRT1 was maximally phosphorylated (**Fig. 5C**).

### Threonine 530 and serine 540 of SIRT1 are targets for cyclinB/Cdk1 *in vitro*

**Figure 6:**
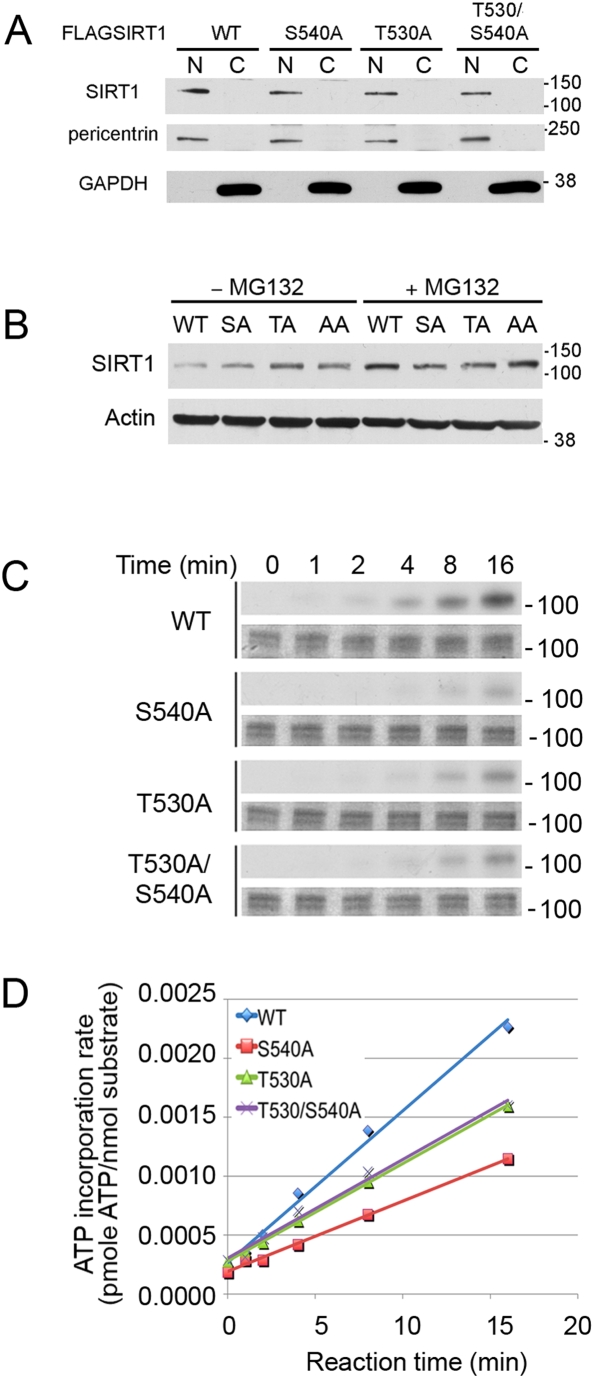
Threonine 530 and serine 540 of SIRT1 are substrates for cyclinB/Cdk1 in vitro. A. Western blot analysis of nuclear (N) and cytoplasmic (C) fractions of cells transfected with FLAG-SIRT1 expression vectors encoding wild type (WT) and mutant SIRT1 proteins. Blots were subsequently reacted with antibodies against pericentrin and GAPDH to demonstrate the purity of the nuclear and cytoplasmic fractions, respectively. B. Western blot analysis of WT and mutant proteins in the presence and absence of the proteasome inhibitor MG132. C/D. *In vitro* kinase assays showing that threonine 530 and serine 540 are substrates for cyclinB/Cdk1 *in vitro*. The results are shown by autoradiograph (top panels) and Coomassie blue stained gel (bottom panels) in (C) and by the plot of ATP incorporation (as measured by scintillation countering) *vs.* time in (D).

To test the hypothesis that Thr530 and Ser540 are cyclinB/Cdk1 substrates directly, we mutated each site to a non-phosphorylatable amino acid (alanine), then tested whether or not the mutant proteins could be phosphorylated by cyclinB/Cdk1 *in vitro*. Mutation of either Ser540→Ala or Thr530→Ala or both had no effect on protein stability or localization (**Fig. 6A–B**). However, compared to wild type, the S540A mutant showed approximately 50% reduction in the incorporation of radioactive ATP and the T530A and T530A/S540A double mutant (AA) showed approximately 35% reduction (**Fig. 6C–D**), indicating that both residues are substrates for cyclinB/Cdk1 *in vitro*. The fact that the radioactive signal decreased in the mutants, but did not go away completely, indicates that other less well-conserved residues that fit the minimal Cdk substrate motif, such as Ser14, Ser27, Ser47 and Ser545 (**Table 2**) may also be substrates for cyclinB/Cdk1 *in vitro*.

### Phosphorylation of SIRT1 at Thr530 and Ser540 influences cell proliferation and cell cycle profiles

**Figure 7:**
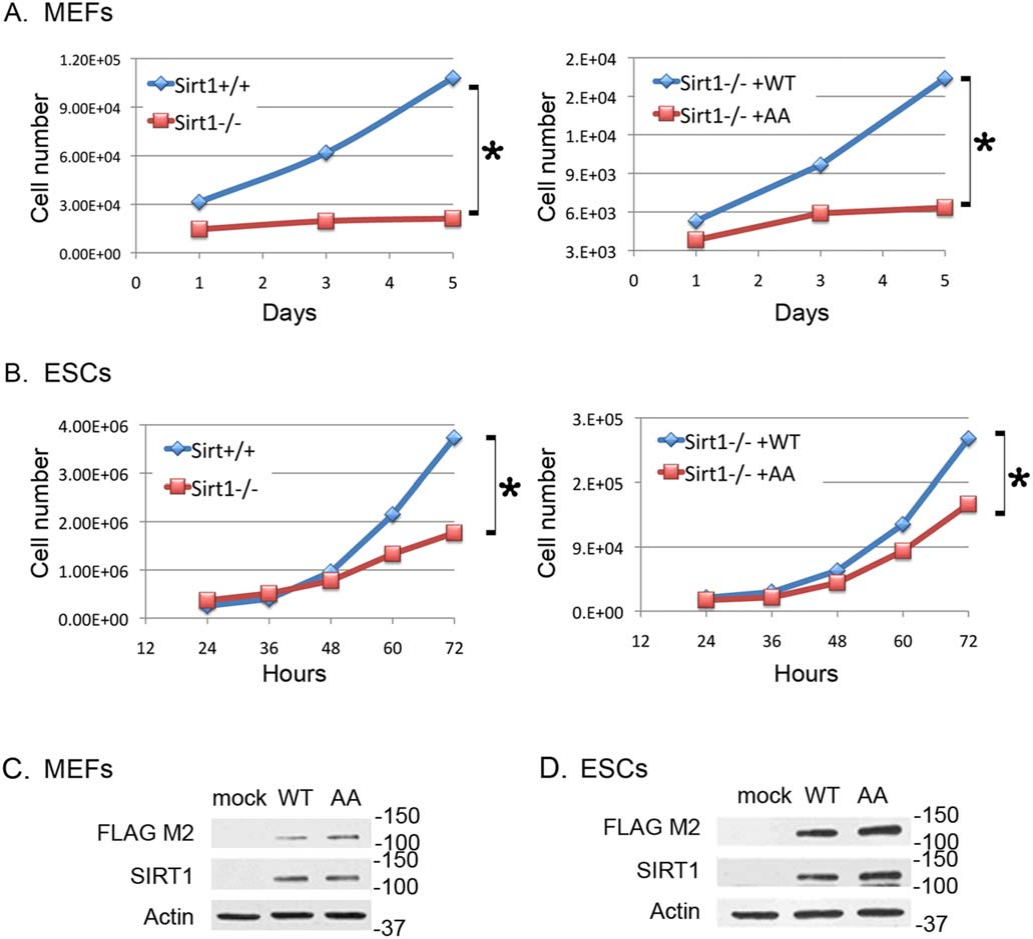
Phosphorylation of SIRT1 at Thr530 and Ser540 affects cell proliferation. Proliferation of Sirt1^+/+^ and Sirt1^−/−^ MEFS (A) and ES cells (B) and in cells transfected with wild-type (WT) or mutant (AA) SIRT1. Representative experiments are shown. Each experiment was performed in triplicate and was repeated a minimum of two times. The differences were significant to varying degrees depending on the experiment and are indicated with an asterisk. Western blot analysis was used to ensure that the levels of expression of wild type or mutant SIRT1 proteins in transfected MEFs (C) and ESCs (D) were equivalent.

To determine if the decline in deacetylase activity associated with global dephosphorylation of SIRT1 was linked to changes in phosphorylation at Thr530 and/or Ser540, we compared the deacetylase activity in preparations of FLAG-SIRT1 with or without the T530A and/or S540A mutations. We found no significant differences in the NAD^+^-dependent deacetylase activity (data not shown). However, another possible function of SIRT1 phosphorylation by cell cycle-regulated kinases was suggested by our previous finding that cell proliferation fluctuates with the level of SIRT1 in cells and tissues (Sasaki 2006). To test the significance of SIRT1 on cell proliferation, we transfected Sirt1-deficient mouse cells with expression vectors for normal FLAG-SIRT1 (WT) or FLAG-SIRT1 with the T530A/S540A mutation (AA). We found that the proliferation defects of Sirt1^−/−^ MEFs (**Fig. 7A**, left-hand panel) and ES cells (**Fig. 7B**, left-hand panel) could be rescued by transfection of WT SIRT1, but not by the T530A/S540A double mutant (**Fig. 7A–B**, right-hand panels). These effects on proliferation were not due to large differences in protein concentration in transfected cells, as shown by western blot in **Fig. 7C–D** for MEFs, and ESCs, respectively.

**Figure 8:**
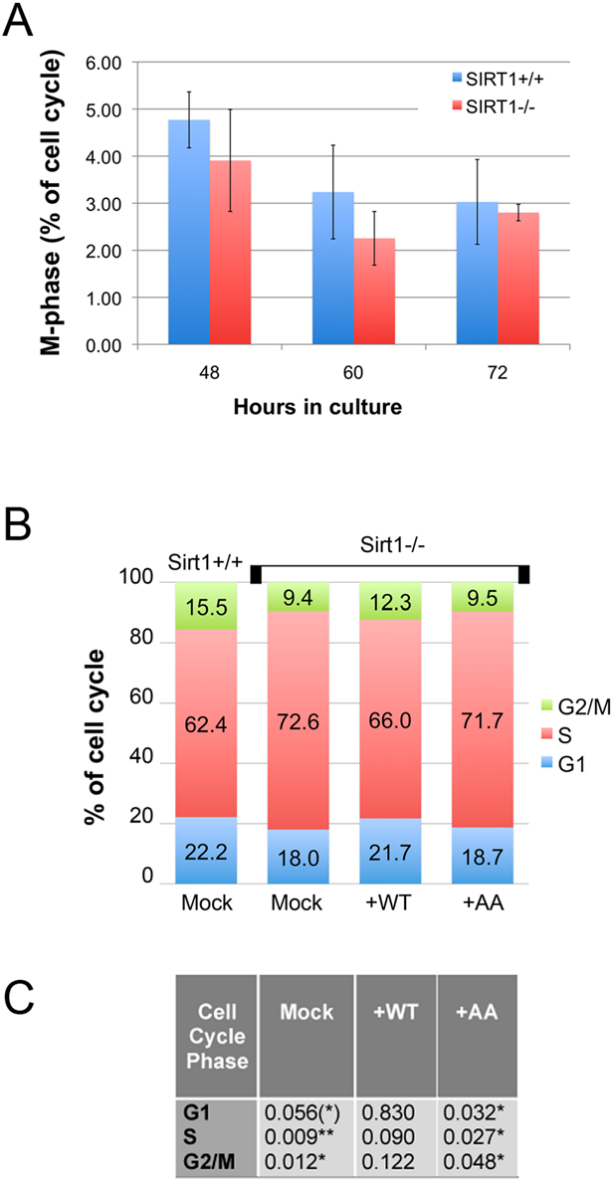
Phosphorylation of SIRT1 at Thr530 and Ser540 is required for normal cell cycle progression. A. Fraction of cells in M-phase of the cell cycle in Sirt1^+/+^ (blue) and Sirt1^−/−^ (red) ES cells at three different time points in culture. The differences were not significant. B. Graphic representation of the fractions of cells in each phase of the cell cycle in Sirt1^+/+^ and Sirt1^−/−^ ES cells transfected with empty vector (mock), WT-FLAG-SIRT1 (WT), or AA-FLAG-SIRT1 (AA) mutant. C. Table of *p*-values comparing mock-transfected Sirt1^+/+^ cells to Sirt1^−/−^ cells transfected with empty vector (Mock), wild-type (WT), or mutant (AA) DNA. *p<0.05; **p<0.01.

To determine which phase of the cell cycle was affected by loss of Sirt1, we compared the cell cycle profiles of wild-type and Sirt1-deficient ES cells. Cells were stained with propidium iodide and an anti-phospho-histone H3 (Ser10) antibody that is detected only during mitosis [31]. Although we expected that phosphorylation by Cdk1, an M-phase kinase, would alter the percentage of Sirt1−/− ES cells in M-phase, there was no difference compared to Sirt1+/+ ES cells (**Fig. 8A**). We did find that Sirt1−/− ES cells have a small, but statistically significant, increase in the proportion of cells in S-phase compared to wild-type (**Fig. 8B**; compare mock-transfected Sirt+/+ and −/− ES cells). To determine if this was due to Cdk1-dependent phosphorylation of mouse Sirt1, we transfected wild-type and Sirt1-deficient ES cells with expression vectors encoding wild-type or mutant (AA) human SIRT1 and again analyzed the cell cycle by FACS. We found that wild-type SIRT1 could rescue the increase in S-phase cells, but the AA mutant could not. The percentages of cells in G1 and G2/M were also significantly different in Sirt1-deficient ES cells transfected with wild-type and mutant SIRT1 (**Fig. 8C**). Rescue of the G2/M-phase difference identified in mock-transfected Sirt1+/+ and Sirt1−/− cells (**Fig. 8B**; green) was due to rescue of G2 deficits by wild-type but not mutant SIRT1, consistent with our finding that M-phase in Sirt1−/− ES cells was normal (**Fig. 8A**). In summary, phosphorylation of SIRT1 by cyclinB/Cdk1 is necessary for progression through the cell cycle, apparently by facilitating entry into G2. Cell proliferation is impaired to the same extent by blocking Cdk1 phosphorylation of SIRT1 and by complete loss of SIRT1. Together with data indicating that dephosphorylation decreases SIRT1 deacetylase activity, these studies reveal a critical role for phosphorylation in regulating SIRT1 function.

## Discussion

In this paper, we provide an explanation for our previous finding that increases in SIRT1 protein when cells are mitotically active are not the result of increased SIRT1 transcription (Sasaki T, *et al.* 2006). We have identified 13 serine and threonine residues in SIRT1 that are phosphorylated, and we demonstrated that one of the kinases that can phosphorylate SIRT1 is the cell cycle-dependent kinase Cdk1. Thus, post-transcriptional modification by phosphorylation appears to play a significant role in increasing the level of SIRT1 protein in cycling cells.

Some of the phosphorylated residues, cyclin-recognition motifs, and Cdk substrate motifs identified in our search are conserved among species. An in depth analysis revealed an interesting aspect of these conserved residues as they relate to organisms that, unlike mammals, consist mainly of post-mitotic cells as adults. The residue that corresponds to Ser540 in human SIRT1 is E (glutamate) in *D. melanogaster* and D (aspartate) in *C. elegans*. S→D and S→E mutations are commonly used as phospho-mimetics, suggesting that, in cells no longer undergoing mitosis, the function of SIRT1 affected by phosphorylation at this residue is essential. Loss of function, for example, might prevent cells from leaving the cell cycle. In fact, previous studies have shown that SIRT1-deficient mouse cells are resistant to replicative senescence (Chua KF, *et al.* 2005). In cells that must retain the ability to proliferate, SIRT1 activity would have to fluctuate according to whether or not the cells are actively dividing. By having that activity subject to cycles of phosphorylation and dephosphorylation regulated by the cell cycle, a feedback loop is set up whereby cyclin/Cdk complexes affect the activity of SIRT1, which in turn regulates proliferation. Thus, we found that wild-type SIRT1, but not the S540A/T530A mutant, was able to rescue the proliferation defect in SIRT1−/− MEFs and ESCs. This is consistent with a recent report demonstrating that forced expression of SIRT1 in human fibroblasts increased their growth rate (Huang J, *et al.* 2008). The fact that SIRT1-deficient mouse cells grow more slowly than their wild-type counterparts could in turn explain why they are resistant to replicative senescence in culture (Chua KF, *et al.* 2005). We would expect (although we did not test this) that enhancing proliferative competence of SIRT1-deficient cells by reintroducing wild-type SIRT1 would have the additional effect of reinstating replicative senescence.

Although this is the first extensive report on SIRT1 phosphorylation, other classes of HDACs are known to be regulated by phosphorylation. The activities and complex formation of HDAC1 and HDAC2 are regulated by phosphorylation [23], [24], and nucleo-cytoplasmic localization of HDAC4, HDAC5, and HDAC7 are regulated by 14-3-3 in a phosphorylation-dependent manner [32], [33]. According to functional motif predictions obtained through the Eukaryotic Linear Motif database [34], Ser26 and Ser27 of human SIRT1 are within interaction motif 2 of 14-3-3, and Ser172 and Ser173 are within interaction motif 3. Thus, it is possible that the interaction of SIRT1 with 14-3-3, which has been demonstrated in *C. elegans* [35], [36], is also regulated by phosphorylation in higher organisms.

The results of our study also imply that SIRT1 might undergo hierarchical levels of phosphorylation, with multiple phosphorylation sites cooperating to maximize subsequent phosphorylation events. According to functional motif predictions obtained through ScanSite (http://scansite.mit.edu) [37], KinasePhos (http://kinasephos.mbc.nctu.edu.tw) [38], and Eukaryotic Linear Motifs (http://elm.eu.org) [34] servers, the 13 phosphorylated residues of SIRT1 identified in this study match target motifs for ATM, CDK5, CK1, CK2, DNA-PK, ERK1, GSK3, IKK and MAPK, in addition to CDK1. Thus, any number of signaling cascades could influence SIRT1 function through the activity of various kinases and their accompanying phosphatases. It was recently reported for HDAC7, for example, that phosphorylation of Ser 155 is a prerequisite for phosphorylation of Ser181 [33] and that dephosphorylation by myosin phosphatase controls its nucleocytoplasmic shuttling and inhibits apoptosis in thymocytes [39]. The large number of potential kinases and the redundancy of kinases that can act on a single site could explain why we observed more robust phosphorylation of SIRT1 using a “mitotic kinase mix” compared to cyclinB/Cdk1 alone (**Fig. 5C**). The addition of other mitotic kinases, such as aurora kinase and polo kinase, present in the mix, significantly enhanced phosphorylation at Cdk1-specific sites.

When SIRT1 was dephosphorylated by treatment with non-specific phosphatases, there was a concomitant decrease in deacetylase activity. Mutation of the Cdk1 phosphorylation sites, however, did not lead to a reduction in deacetylase activity, at least as measured by the *Fluor-de Lys* deacetylase assay. One possible reason for this is that additional Cdk phosphorylation sites, such as Ser14, Ser26/27, Ser47 and Ser545, or any one of the other 11 phospho-residues, may have to be mutated before loss of Cdk1 phosphorylation will affect deacetylase activity. This could explain why the AA mutant showed a comparable amount of radioactive ATP incorporation in the *in vitro* kinase assay (**Fig. 6B**). Another possibility is that phosphorylation of certain residues may have positive influence on deacetylase activity, while others may have a negative or no influence. It should be noted that even relatively complete dephosphorylation by non-specific phosphatases did not totally abolish deacetylase activity (**Fig. 2**). Considering the fact that all the phosphorylation sites are located outside of the conserved catalytic domain, it is also possible that phosphorylation of these residues could affect enzyme activity indirectly. Based on predictions of 3D structure, Thr530 and Ser540 reside in the hinge region of SIRT1 between the conserved core domain and the C-terminal domain. As is clear from **Fig. 1C**, phosphorylation of these residues could alter the accessibility of substrate molecules to the catalytic groove of the deacetylase. This might be why there were significant differences in the effects of wild-type and mutant SIRT1 on biological phenotypes, such as cell proliferation and cell cycle profiles, despite there being no detectable difference in deacetylase activity between them in vitro. It should be noted that a similar result was observed following mutation of the Cdk1 site in SIRT2 [40], a cytoplasmic isoform of SIRT1. SIRT2 is phosphorylated on multiple residues during the G2/M transition and is dephosphorylated by CDC14A and CDC14B phosphatases [40], [41]. Thus, it appears that multiple sirtuins may be regulated by cell cycle-dependent kinases.

The targets that SIRT1 deacetylates in the context of mitosis remain undefined. CyclinB/Cdk1 kinase activity is tightly regulated and increases during M phase of the cell cycle, yet substrates vary and include proteins that regulate DNA replication, mitosis, spindle assembly, actin polarization, and other processes in yeast [42]. The identification of the molecular targets that are affected by SIRT1 phosphorylation, especially at sites modified by cell cycle-dependent kinases like Cdk1, will help to elucidate how changes in the level and activity of SIRT1 affect diverse processes such as growth and longevity in organisms ranging from single-celled yeast to mammals.

## Materials and Methods

### Cell culture

Sirt1^−/−^ and Sirt1^+/+^ ES cells (gifts from Dr. F. Alt, Harvard) were cultured on gelatin-coated plates in DMEM supplemented with 15% FBS, L-glutamine, non-essential amino acids, nucleosides, β-mercaptoethanol, murine leukemia inhibitory factor (LIF) (all from Millipore, Billerica, CA) and penicillin/streptomycin (Invitrogen, Carlsbad, CA). ES cells were cultured in the absence of feeder cells to get an accurate number for cell counting. 293T cells were purchased from ATCC (Manassas, VA). 293T cells were cultured as previously described [9]. For protein stability experiments, cells were cultured for 3 hours in the presence of the proteasome blocker MG132.

### Expression vector construction and transfection

SIRT1 mutant expression vectors were generated by PCR mutagenesis using the SIRT1-expression vector pcDNA4/TO/FLAG-SIRT1 (a gift from Dr. D. Reinberg, NYU) [43] as a template. The CMV promoter of the pcDNA4/TO/FLAG-SIRT1 vector was replaced with the Pgk promoter at MluI/EcoRV sites to allow the expression of FLAG-SIRT1 in ES cells. For the empty pPGK vector, the pPGK-FLAGSIRT1 vector was digested with EcoRV and PmeI to remove FLAG-SIRT1 cDNA, and then blunt-end ligated. Plasmids were transfected using Lipofectamine 2000 (Invitrogen) following the manufacturer's protocol.

### Protein preparation and immunoprecipitation

Total cell lysates were prepared as previously described [9]. Immunoprecipitations of total cell lysates were performed with FLAG-M2 beads (Sigma-Aldrich), or anti-SIRT1 N-terminal polyclonal antibody (Millipore #07-131), anti-SIRT1 C-terminal polyclonal antibody AS-16 (Sigma-Aldrich #S5313) and protein G agarose (Millipore) for 2 hours or overnight. After washing 3 times with lysis buffer, SDS-PAGE and western blot analysis were performed. FLAG-SIRT1 protein was purified by incubating Benzonase-treated (Sigma-Aldrich) total cell lysates with anti-FLAG M2 beads for 2 hours or overnight, then washed 3 times with wash buffer (10% glycerol, 50 mM Tris pH 7, 150 mM NaCl), then eluted with FLAG peptide (Sigma-Aldrich). Further gel filtration was performed using HiLoad 6/16 Superdex 200 column and AKTA FPLC system (GE Healthcare, Piscataway, NJ) when necessary. Samples were concentrated by centrifugation through Microcon YM-10 filters (Millipore). GST-SIRT1 protein was purified as previously described using BL21-CodonPlus competent cells (Stratagene, La Jolla, CA) and the GST-SIRT1 expression vector pGEX2TK-SIRT1 (a gift from Dr. J. Smith, UVA) [44]. The purity of the proteins was tested by western blot and Coomassie blue staining, and the activities were measured using the *Fluor-de-Lys* deacetylase assay kit (Biomol International, Plymouth Meeting, PA). Antibodies used for the co-IP of endogenous proteins are as described for western blot analysis below.

### Western blot analysis

Samples were separated by SDS-PAGE using 10% or 4–20% Tris-HCl gels and transferred using standard protocols. To detect the antigen-antibody complexes, we used Western Pico or Femto chemiluminescence substrate (Pierce, Rockford, IL). The following antibodies were used: Peroxidase-conjugated AffiniPure Goat-anti-Mouse IgG and biotinylated anti-Mouse light chain (Jackson ImmunoResearch, West Grove, PA, #115-035-100, 1∶10,000), Peroxidase-conjugated AffiniPure Goat anti-Rabbit IgG (Jackson ImmunoResearch #110-035-144, 1∶10,000), anti-Cdc2 (Cdk1) mAb (Santa Cruz Biotechnology, Santa Cruz, CA, sc-8395, 1∶1,000), anti-Cdk2 pAb (Abcam, Cambridge, MA, ab7954, 1∶1,000), anti-Cdk4 pAb (Abcam ab2945, 1∶1,000), anti-Cdk6 mAb (Abcam ab3126, 1∶1,000), anti-cyclinA pAb (Abcam ab7956, 1∶1,000), anti-cyclinB1 pAb (Abcam ab7957, 1∶1,000), anti-FLAG M2 mAb (Sigma-Aldrich F3165, 1∶2,000), anti-SIRT1 N-terminal polyclonal antibody (Millipore #07-131, 1∶1,000), anti-SIRT1 C-terminal polyclonal antibody AS-16 (Sigma-Aldrich #S5313, 1∶1,000), anti-SIRT1 monoclonal antibody 2G1/F7 (Millipore #05-707, 1∶1,000), anti-phospho serine Cdk substrate antibody (Cell Signaling Technology, Danvers, MA, #2324, 1∶2,000), anti-GAPDH antibody (Abcam ab8245, 1∶5,000), and anti-pericentrin antibody (Covance, Richmond, CA, PRB-432C, 1∶300). For **Figure 4b**, the ODYSSEY infrared imaging system (LI-COR, Lincoln, NE) was used to quantitate the percentage of phosphorylated SIRT1 in λppase-treated samples.

### Comparative analysis of SIRT1 amino acid sequences

Amino acid sequences of SIRT1 from various species were obtained from the NCBI database, and subjected to the BLAST 2 Sequence algorithm (http://www.ncbi.nlm.nih.gov/BLAST/) against the human SIRT1 sequence. The following sequences were used for the analysis: *Homo sapiens* (human, NP_036370), *Pan troglodytes* (chimpanzee, XP_521490), *Macaca mulatta* (macaque monkey, XP_001087854), *Canis familiaris* (domestic dog, XP_546130), *Bos taurus* (cow, XP_869911), *Mus musculus* (mouse, AAR23928), *Rattus norvegicus* (rat, XP_001080493), *Gallus gallus* (chicken, NP_001004767), *Drosophila melanogaster* (fruit fly, AAC79684), *Caenorhabditis elegans* (round worm, CAA94364), *Schizosaccharomyces pombe* (fission yeast, CAG47122) and *Saccharomyces cerevisiae* (budding yeast, NP_010242).

### ProQ Diamond phospho-protein staining

After protein samples were separated by SDS-PAGE and transferred to nitrocellulose membrane, the membrane was stained with Pro-Q Diamond phosphoprotein staining kit (Invitrogen P-33356) following the manufacturer's protocol.

### Phospho-peptide mapping by mass spectrometry

Phospho-peptide mapping was done as previously described [45]. The gel band containing FLAG-SIRT1 was identified by silver stain, cut out, and the peptides generated by trypsin treatment. Peptides were analyzed using the LC-MS system.

### Structure prediction for human SIRT1

Protein sequence information for human SIRT1 (NP_036370) was obtained from the PubMed protein database. This protein sequence was used to derive a full chain protein structure prediction on the Robetta server (Baker Laboratory, http://robetta.bakerlab.org/). The Robetta server subjected the SIRT1 sequence first to the Ginzu protocol that determined putative domain structures and then modeled those domains either by homology or by *ab initio* modeling. Out of ten model predictions, the best fitting was chosen to illustrate the full chain SIRT1. VMD1.8.6 (http://www.ks.uiuc.edu/Research/vmd/) [46] was used for graphical illustration of the predicted structure and the phosphorylation sites.

### Phosphatase preparations and treatments

Solutions of calf intestinal phosphatase (New England Biolab, Ipswich, MA) or lambda phosphatase (a gift from Dr. D. Brautigan, UVA) were prepared and incubated with FLAG-SIRT1 samples at 1∶1 volume for 1 hour at 37°C unless otherwise indicated. Phosphatase activity was blocked by adding phosphatase inhibitor cocktails I & II (Sigma-Aldrich) or 10 mM Na_3_VO_4_ (Sigma-Aldrich).

### *In vitro* kinase assay

Kinase reactions were performed in 20 mM Tris HCl (pH 7.5), 1 mM MgCl_2_, 25 mM KCl, 1 mM DTT, and 40 µg/mL BSA, with 100 µM of ATP spiked with γ-^32^P-ATP. CyclinB/Cdk1 purified from *Xenopus* was used with 5–10 pmole of substrate. Samples were harvested at each time point, and reactions quenched with 6×-sample buffer. Samples were separated by SDS-PAGE and stained with Bio-Safe Coomassie (Biorad). Gels were photographed with ChemiImager Ready (IMGEN technologies, Alexandria, VA). After autoradiography, each protein band was cut out of the gel and the radioactivity in the sample measured using a Beckman LS 6000SE scintillation counter (Beckman Coulter, Fullerton, CA). “Mitotic kinase mix” (a gift from Dr. D. Brautigan, UVA) was prepared according to the protocol described in reference [30].

### *Fluor-de Lys* deacetylase assay

*Fluor-de Lys* deacetylase assay kit (Biomol International) was used to analyze deacetylase activities in various SIRT1 preparations, using a slight modification of the protocol recommended by the supplier to adjust for the detection range of the fluorometer. Nicotinamide was added to the reaction at the concentration of 2 mM when necessary. The final fluorescence was measured in a Fluoroskan Ascent FL fluorometer (Thermo Fisher Scientific, Waltham, MA).

### Cell proliferation assay

Lipofectamine 2000 (Invitrogen) was used to transfect 1×10^6^ ES cells with 2 µg of empty vector or expression vectors for FLAG-SIRT1 with or without the T530A and S540A double mutation. All vectors carried the PGK-promoter to support expression in ES-cells. 24 hours after transfection, 2×10^4^ cells/well were re-plated onto gelatin-coated 12-well plates, and cells were counted 24, 36, 48, 60, and 72 hours after replating. Each group consisted of triplicates and the experiment was done three times, and the average and the standard error of the number of cells in each well were calculated. For MEFs, 5×10^4^ cells/well at passage 3 (split at 1∶4 at each passage, used prior to immortalization) were transfected with expression constructs driven by the CMV promoter, and done in triplicate.

### Cell cycle analysis

Cells were harvested by trypsinization, washed with phosphate-buffered saline (PBS), and fixed in 70% ethanol at a concentration of 10^6^ cells per ml at −20°C for 2 hours or overnight. After fixation, cells were stained with propidium iodide (Sigma-Aldrich) and anti-phospho histone H3 (Ser10) mitosis marker antibody (Millipore, 1∶1,000) followed by Alexa 488-conjugated goat anti-rabbit immunoglobulin G antibody (Invitrogen, 1∶2,000), as previously described [31]. Analyses were done at the University of Virginia Flow Cytometry Facility and cell cycle distributions were analyzed using ModiFit LT version 3.1 software (Verity Software House, Topsham, ME). The number of cells in the G2 phase of the cell cycle was calculated by subtracting the number of cells in M phase (determined by phospho-H3 staining) from the number of cells in G2/M phase (determined by PI staining).
